# RNA therapy: rich history, various applications and unlimited future prospects

**DOI:** 10.1038/s12276-022-00757-5

**Published:** 2022-04-19

**Authors:** Young-Kook Kim

**Affiliations:** Department of Biochemistry, Chonnam National University Medical School, Hwasun, Jeollanam-do 58128 Republic of Korea

**Keywords:** Drug discovery, Diseases, Drug development

## Abstract

RNA therapy refers to the treatment or prevention of diseases using RNA-based molecules. The recent advent of a series of effective messenger RNA-based vaccines in response to the COVID-19 pandemic has reignited research interest in RNA therapy. Based on the accumulated results of long-term research in the field of RNA therapy spanning several decades, therapeutic agents for various diseases are being rapidly developed. These therapeutics tend to target diseases that cannot be treated with other conventional drug groups, and several clinical studies are underway for a variety of RNA-based therapeutics against various incurable diseases. This review describes the history of several important discoveries in RNA biology and their impact on key developments in RNA therapy as well as the advantages of RNA therapy. In addition, it describes the action mechanisms and examples of drugs approved for RNA therapy. Finally, this review discusses methods for RNA drug delivery to target organs and cells. Given that RNA therapy is expected to advance and play an integral role in the development of novel therapeutic agents for human diseases in the future, this review is designed to offer an updated reference point for researchers in this field.

## RNA therapies: optimising drug delivery for treating the untreatable

RNA-based therapies should improve the lives of many people affected by difficult-to-treat diseases, provided novel drug delivery methods are fully explored and optimized. Young-Kook Kim at Chonnam National University Medical School in Hwasun, Korea, reviewed the current status of RNA therapies, particularly given recent successes in developing messenger RNA vaccines for COVID-19. RNA therapeutics work by manipulating the expression and activity of specific target molecules, providing the means to treat diseases that do not respond to conventional drug types. Such therapies can be tweaked to cover a wide range of different forms of RNA and protein, and could open the way to personalized medicines and treatments for rare diseases. However, RNA-based drugs are larger molecules than other therapeutics, making targeted delivery within the body more difficult. Kim suggests that ensuring effective RNA drug delivery should be a paramount focus for future research.

## Introduction

RNA therapy is a term used to describe the use of RNA-based molecules to modulate biological pathways to cure a specific condition. In general, the RNA sequence is the key to manipulating the expression or activity of its target molecules. Once the nucleic acid chemistry and the delivery method are established, the production of RNA-based drugs for a new target can be achieved in a relatively short period using these pre-established methodologies. It is this strength that has made messenger RNA (mRNA) therapy, a subcategory of RNA therapy, the most important and efficient means for developing novel vaccines used to combat coronavirus disease 2019 (COVID-19). The unprecedented efficacy of these vaccines has reignited interest in RNA-based therapy, but these successes were possible only due to the preceding 30 or more years of research and development in this field.

Here, I aim to introduce the key discoveries underlying the development of current RNA-based therapeutic technologies. I then discuss the key strengths of these RNA-based therapies compared to other drug compounds, including small molecules and antibodies, and describe the different technologies included in the term RNA therapies. This will include a description of antisense single-stranded RNA, double-stranded small interfering RNAs, aptamers, and mRNAs, their mechanism of action, and a discussion of the representative drugs in each category. This categorization is based on my previous paper discussing a similar topic^1^. However, this review will include the details of the molecular mechanisms of approved RNA-based drugs or those drugs in the last stage of clinical trials. This paper will also discuss RNA-based vaccines, which were not covered in the previous paper. Finally, the delivery methods for RNA-based drugs, one of the most actively researched questions in RNA therapy, will be presented.

## Historical overview of RNA therapy

**Fig. 1:**
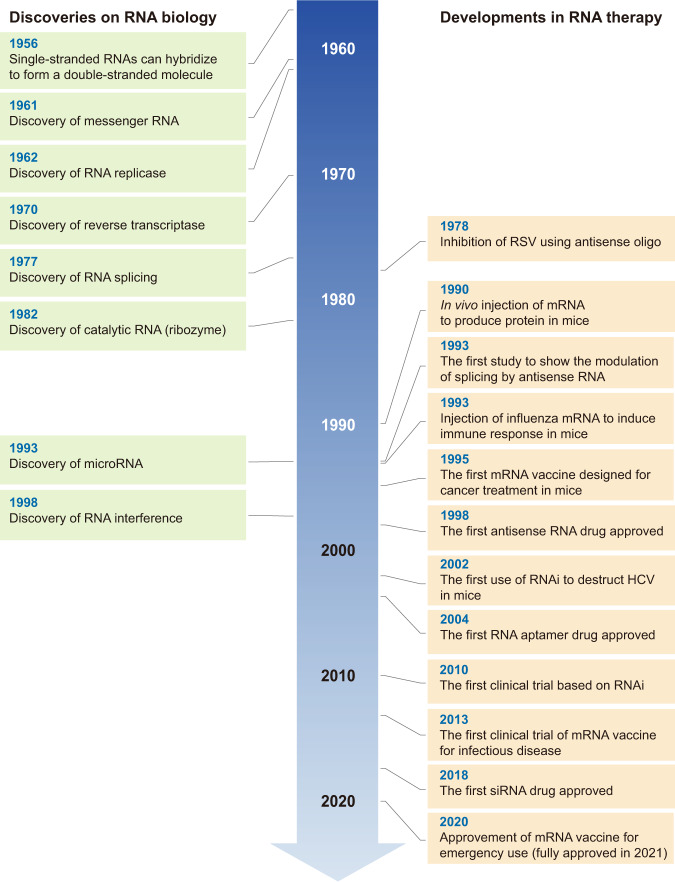
The historical timeline of important discoveries in RNA biology and key developments in RNA therapy. See text for details.

Numerous discoveries have established RNA therapy as an indispensable technology for treating human disease (Fig. 1). RNA was first described as a key player in the flow of genetic information by Crick in his study “Central Dogma of Molecular Biology”^2^ and was later confirmed by the discovery of mRNA, which highlighted the importance of these molecules as key messengers in the translation of genetic information^3,4^.

However, there was a second equally important, but less commonly discussed, discovery critical to the field of RNA, which was the discovery that two RNAs can base-pair with each other. Although this information is now taken for granted, early RNA scholars did not believe that RNA could produce a double helix structure. However, in 1956, Rich and Davies published the first nucleic acid hybridization reactions, which demonstrated that RNA could form a similar configuration to DNA based on the same complementarity in its base-pairing^5^. This discovery formed the basis for the subsequent discovery of microRNAs (miRNAs) in 1993 and of RNA interference in 1998, in which the production of RNA duplexes is a key step in RNA silencing^6–8^.

However, the first application of RNA base-pairing for therapeutic purposes was first described by Stephenson and Zamecnik in 1978^9^, with the design of an antisense oligonucleotide targeting the 35S RNA sequence of the Rous sarcoma virus (RSV) to inhibit viral replication. Approximately 20 years later, the first drug using an antisense oligonucleotide was approved by the United States Food and Drug Administration (US FDA) for the treatment of cytomegalovirus retinitis^10^.

RNA splicing, the posttranscriptional step joining exons and removing introns from initial RNA transcripts, was first described in 1977^11,12^ and is now a well-known mechanism for disease, with splicing variations being linked to various human diseases^13,14^. The restoration or modulation of these splicing defects is almost impossible using conventional small molecule-based drugs but can be accomplished using RNA-based drugs, especially antisense oligonucleotides. In fact, a study from 1993 was the first to note that alternative splicing can be modulated by the application of antisense oligos^15^. In this study, a set of antisense oligonucleotides targeting the splice sites and branch points of thalassemic pre-mRNA were used to correct its abnormal splicing and reduce symptoms. This paper now serves as one of the cornerstones in the development of novel therapies for several difficult-to-treat neurological diseases described below.

In contrast to the long history of the development of antisense oligo-based drugs, it took a relatively short time to proceed from the discovery of small interfering RNAs (siRNAs) to the utilization of these molecules as drugs. RNA interference (RNAi) was first characterized in a seminal paper in 1998, which demonstrated that treating *Caenorhabditis elegans* embryos with a mix of sense and antisense RNAs led to the potent and specific inhibition of targeted endogenous mRNAs^8^. Because RNAi is simple and powerful, it was rapidly adopted by the scientific community as a whole and applied extensively in a very short period. One example of this is a paper from 2002 that described the use of RNAi to inhibit hepatitis C virus replication in mice, which resulted in the widespread evaluation of RNAi for therapeutic purposes^16^. This facilitated the first clinical trials based on RNAi technologies in 2010, where a siRNA targeting the M2 subunit of ribonucleotide reductase was used to treat a patient with widespread melanoma^17^. This trial reported the successful cleavage of the target mRNA when the siRNA was delivered using a targeted nanoparticle delivery system. Following this trial, several siRNA-based drugs were evaluated for a diverse array of diseases, with the first siRNA drug for patients with hereditary transthyretin-mediated amyloidosis being approved in 2018^18^.

Despite the critical identification of mRNA as a messenger for genetic translation in 1961, it took nearly 30 years for researchers to establish a way to exploit this molecule for therapeutic purposes. In particular, the discovery of several RNA-related enzymes a few decades ago has facilitated the broader development of our current mRNA-based therapies. In the early 1960s, RNA-dependent RNA polymerase (RdRp; also known as RNA replicase) was first discovered in a series of studies on viruses (mengovirus and poliovirus)^19,20^, in which the authors found that these viruses were not sensitive to an inhibitor of DNA-dependent RNA polymerase, actinomycin D. Currently, RdRp is used as an essential factor in the production of self-amplifying RNA vaccines^21^. Another key enzyme, reverse transcriptase, was also first identified in viruses and is the essential enzyme in the life cycle of most retroviruses. In practice, researchers exploit this enzyme to clone the complementary DNA template to facilitate the production of the desired mRNA. Thus, the discovery of reverse transcriptase in 1970 has been critical to the manufacture of almost all mRNA-based drugs^22,23^.

Attempts to produce specific proteins via the introduction of exogenous mRNA began in the 1990s. In 1990, Wolff and colleagues injected the mRNAs for several reporter genes directly into the skeletal muscle of mice^24^ and observed persistent gene expression in these tissues despite the lack of a delivery system. This later led to the evaluation of mRNA transcripts as a vaccine, which was an approach first tested in 1993^25^. Here, the researchers synthesized the mRNAs for the influenza nucleoprotein using in vitro transcription and encapsulated them into liposomes. These were then injected into mice and were shown to elicit the production of virus-specific cytotoxic T lymphocytes in these animals. Subsequently, Conry *et al*. designed the first mRNA vaccine for the treatment of cancer in 1995. They reported that their vector, which expressed human carcinoembryonic antigen (CEA), could safely be injected into CEA-expressing tumor cells in a mouse model^26^. However, it took a long time for these animal experiments to yield sufficient data to allow this technology to move to human clinical trials. In 2008, Weide and colleagues reported the first set of results for their clinical trial using mRNA vaccines in patients with metastatic melanoma^27^. These studies used protamine-protected mRNAs coding for tumor-associated antigens, and the results showed that the delivery of these vectors induced an increase in vaccine-directed T cells^28^. The first clinical trial of an mRNA vaccine against infectious disease was conducted in 2013 (NCT02241135) and was designed to evaluate the efficacy of a novel rabies vaccine designed to deliver an mRNA encoding its glycoprotein. These evaluations revealed that treatment with this vaccine elicited functional antibodies targeting these viral antigens, demonstrating a clear proof of principle for this technology^29^. Finally, the first mRNA-based vaccine against an infectious disease, severe acute respiratory syndrome coronavirus 2 (SARS-CoV-2), was approved by the majority of the world in 2020. This brief description demonstrates the rich history of RNA therapeutics and shows that recent achievements in mRNA vaccines were made possible by the results of many studies completed over more than a four-decade span.

## Advantages of RNA therapy

RNA-based drugs are known to exhibit a wide variety of advantageous traits, making them ideal candidates for the development of various novel therapeutic strategies. Here, I will discuss some of the major advantages of these technologies over more conventional therapeutic compounds.

### Targeting undruggable targets

**Fig. 2:**
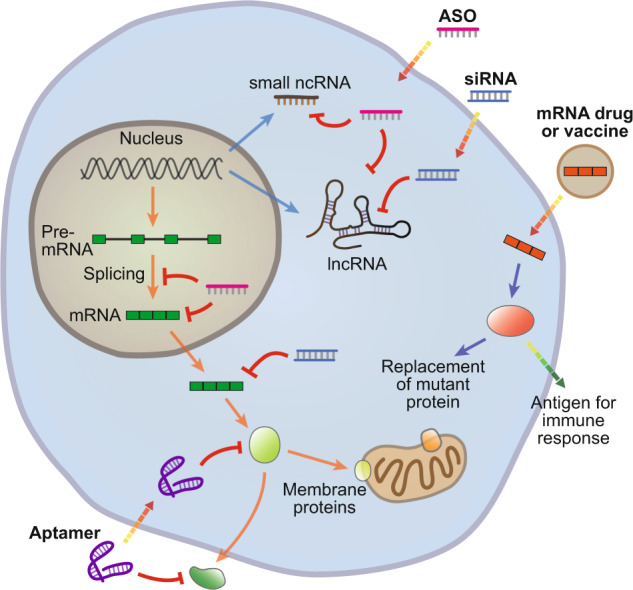
Diverse cellular molecules can be targeted by RNA therapy. RNA-based drugs can target various steps involved in the expression of both protein-coding and noncoding genes. Splicing can be modulated by antisense oligonucleotides (ASOs), and mature messenger RNAs (mRNAs) can be targeted by ASOs or small interfering RNAs (siRNAs). In addition, noncoding RNAs (ncRNAs), including small ncRNAs and long ncRNAs (lncRNAs), can be suppressed by ASOs or siRNAs. Protein function can be modulated by aptamer binding. Finally, exogenous mRNAs can be used to introduce specific proteins into cells to replenish a deficient enzyme or act as antigens to elicit a targeted immune response.

One of the greatest advantages of RNA-based drugs is their ability to target almost any genetic component within the cell, many of which may be considered undruggable using other technologies, including both small molecules and antibodies (Fig. 2). In the case of noncoding RNAs, especially small RNAs, few characteristics distinguish them from one another apart from their RNA sequence. RNA-based drugs, including antisense RNA and siRNA, operate via sequence-specific binding of their target molecules, suggesting that these drugs are likely to be the most effective when targeting noncoding RNAs. Because there are more noncoding RNAs than proteins in the human genome^30^, and because recent studies have confirmed the essential roles of these noncoding RNAs in the pathogenesis of various human diseases^31–33^, the importance of RNA drugs that can target these molecules will only increase.

Moreover, previous studies have suggested that less than one-third of all human proteins can be effectively targeted by small molecules^34,35^. Since many proteins have similar structures, it is not easy to directly target a single specific target protein. In addition, membrane-integrated proteins are even harder to target using small molecules or antibodies due to their limited interactions with the cytoplasm. However, because RNA-based drugs can block the biogenesis of these proteins, they may be better suited to inhibiting their production, improving therapeutic efficacy.

### Fast production

In general, the development process to produce novel small molecule or antibody-based drugs takes years. However, once the chemical structure of the RNA and the means of delivery into the body are established, RNA-based drugs can be designed and synthesized rapidly for clinical tests. For example, if a siRNA-based drug is developed to treat a disease caused by the overexpression of a gene in a specific organ, simply changing the sequence of the siRNA has the potential to treat other diseases within the same organ. This is why siRNA drugs using liver cell-specific conjugates are under widespread development as therapeutic agents for various liver metabolism-related diseases^36^.

One of the reasons that we were able to curb the COVID-19 pandemic and significantly reduce the severity of the disease in infected people was that vaccines against COVID-19 were developed so quickly. Both BioNTech/Pfizer and Moderna were able to design mRNA sequences for their vaccine candidates, apply them in animal experiments, and conduct clinical trials within a short period of time after the sequence of the novel coronavirus was released from China^37,38^. These companies are able to quickly adapt this technology, as their production chain does not vary, making these items ideal for pandemic preparedness.

### Long-term effect

Although natural RNAs are easily degraded by native nucleases, the stability of RNA greatly increases when various modifications are applied to their synthesis^39^. In addition, when these RNAs are encapsulated within carriers such as liposomes, the RNA is efficiently protected from attack by nucleases following systemic administration^40^, improving its longevity. The long-acting effect of RNA-based drugs is exemplified by the recently developed drug inclisiran. This drug is a synthetic siRNA targeting proprotein convertase subtilisin/kexin type 9 (PCSK9) mRNA. Notably, the silencing effect of inclisiran lasts more than six months after only a single injection^41^, allowing significant increases in the administration intervals and potentially reducing toxicity compared to small molecule-based drugs whose effectiveness is halved within a few days.

### Useful for rare disease

Pharmaceutical companies are often reluctant to produce new drugs for very rare diseases because the investment is not warranted by the potential profit. However, in the case of RNA-based drugs, once the chemistry of the RNA and its delivery system are optimized, the cost of developing novel variants of these drugs for new diseases is greatly reduced. One important example of this is the recent development of a splicing-modulatory antisense RNA drug designed to treat a single patient with a rare genetic disease^42^, which would have been impossible using other technologies. Because the technologies related to the development of this antisense drug were well established, it was possible to develop this unprecedented therapy due to reduced processes and development costs.

### No risk of genotoxicity

RNA therapy also has no significant genotoxic effects compared to DNA therapy. In DNA-based therapies, the DNA molecule is delivered to the cells using a viral vector, and there is a possibility that this vector may integrate into the genome and cause a mutation. This potential risk can be avoided when using RNA instead of DNA.

## Types of RNA therapy

RNA-based drugs can be classified into four unique categories based on their structural characteristics and mode of action (Table 1). Here, I review these specifics and discuss some of the major therapeutics developed or under development in each class.

**Table 1:** The working mechanisms and target diseases of approved RNA drugs described in this paper.

RNA drug	Brand name	Approved year	Action mechanism	Target disease
**Antisense oligonucleotides**				
Fomivirsen	Vitravene	1998	Inhibition of the translation of viral mRNA encoding IE2 protein	CMV retinitis
Mipomersen	Kynamro	2013	Induction of the degradation of APOB mRNA	Familial hypercholesterolemia
Nusinersen	Spinraza	2016	Induction of exon inclusion in SMN2 mRNA	Spinal muscular atrophy
Eteplirsen	Exondys 51	2016	Induction of exon skipping in DMD mRNA	Duchenne muscular dystrophy
Inotersen	Tegsedi	2018	Induction of the degradation of TTR mRNA	Hereditary transthyretin amyloidosis
Golodirsen	Vyondys 53	2019	Induction of exon skipping in DMD mRNA	Duchenne muscular dystrophy
**Small interfering RNAs**				
Patisiran	Onpattro	2018	RNA interference-mediated cleavage of TTR mRNA	Hereditary transthyretin amyloidosis
Givosiran	Givlaari	2019	RNA interference-mediated cleavage of ALAS1 mRNA	Acute hepatic porphyria
Lumasiran	Oxlumo	2020	RNA interference-mediated cleavage of HAO1 mRNA	Primary hyperoxaluria type 1
Inclisiran	Leqvio	2021	RNA interference-mediated cleavage of PCSK9 mRNA	Hypercholesterolemia
**RNA aptamers**				
Pegaptanib	Macugen	2004	Antagonistic binding to VEGF protein	Age-related macular degeneration
**Messenger RNAs**				
Tozinameran	Comirnaty	2020	Induction of immune response by producing the spike protein of SARS-CoV-2	COVID-19
Elasomeran	Spikevax	2020	Induction of immune response by producing the spike protein of SARS-CoV-2	COVID-19

### Antisense oligonucleotides

Antisense oligonucleotides modulate the expression of target RNAs via sequence-specific binding, and although the structure of these antisense oligonucleotides is determined primarily by their specific sequence, their chemistry can be modulated to produce novel effects. These modifications can endow antisense oligonucleotides with increased specificity and stability^39^.

Fomivirsen was the first antisense oligonucleotide drug approved by the FDA in 1998 and is used in the treatment of cytomegalovirus retinitis, especially in patients coinfected with human immunodeficiency virus (HIV). Fomivirsen binds to the cytomegalovirus (CMV) mRNA encoding the immediate-early 2 protein, which is essential for CMV replication^10^. However, this drug was withdrawn by the FDA in 2001 because the antiretroviral therapies used in patients with HIV significantly reduced the demand for fomivirsen.

**Fig. 3:**
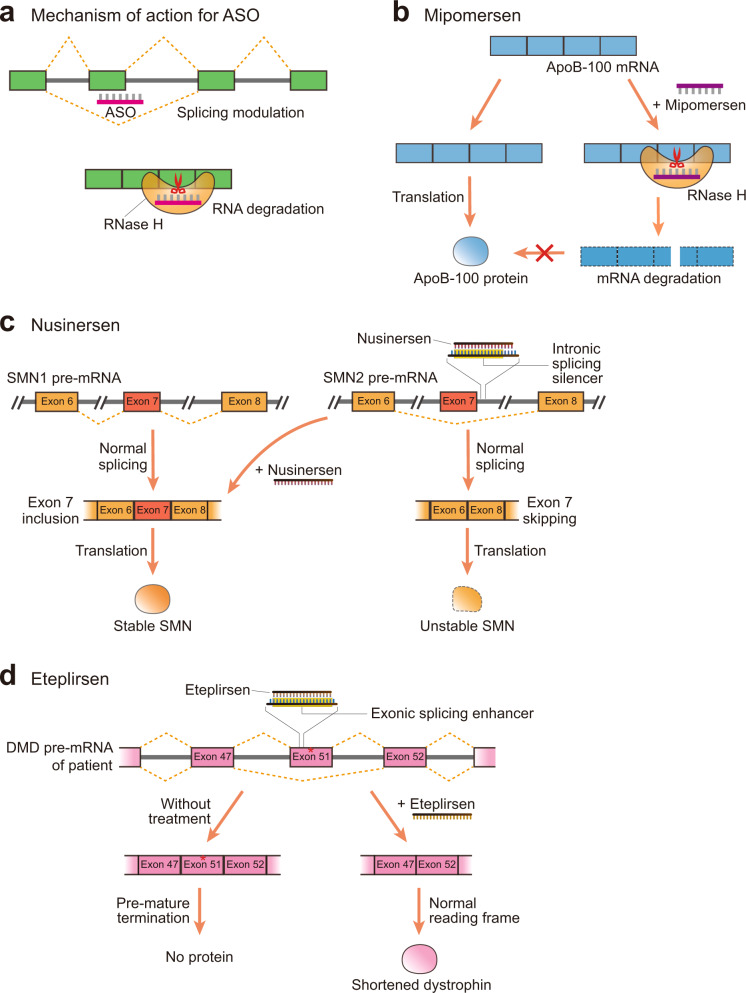
Antisense oligonucleotide-based RNA drugs. **a** Primary mechanism of action for antisense oligonucleotide (ASO)-based RNA drugs in human cells. ASOs can modulate splicing or induce RNase H-mediated degradation of the target mRNA. **b** Mipomersen, a drug used in the treatment of homozygous familial hypercholesterolemia, induces the degradation of apolipoprotein B-100 (ApoB-100) mRNA. **c** Nusinersen binds to the intronic splicing silencer element in the intron of survival motor neuron 2 (SMN2) pre-mRNA, which blocks the exon 7 skipping associated with SMN type diseases. Consequently, SMN2 mRNA is spliced like SMN1 mRNA, resulting in the production of stable SMN proteins. **d** In some patients with Duchenne muscular dystrophy, a mutation at exon 51 of dystrophin (DMD) mRNA induces premature termination of translation, preventing the production of functional protein. Eteplirsen binds to the exonic splicing enhancer in this exon and induces exon 51 skipping. This results in the production of smaller amounts of fully functional proteins.

Antisense oligonucleotides use several diverse mechanisms of action, but approved antisense oligonucleotide drugs can be divided into two broad categories based on their mechanism (Fig. 3a). The first group induces the cleavage of a target mRNA by binding to the target sequence. These antisense oligonucleotides are often modified to include DNA-based central sequences surrounded by chemically modified RNA. Once these antisense oligonucleotides form a duplex with their target RNA, their central region produces a DNA-RNA hybrid that is recognized by RNase H. RNase H then cleaves the RNA sequence between the DNA and RNA duplex, inducing the degradation of the target RNA. Because these therapeutics rely on the activity of RNase H, which is active in both the nucleus and cytoplasm, these drugs can be used to target noncoding elements^43,44^, providing an advantage in some situations compared to siRNA-based drugs that operate primarily in the cytoplasm^45^.

Several antisense oligonucleotide drugs using this type of cleavage have been approved by the US FDA, including mipomersen and inotersen (antisense oligonucleotides contain an -rsen suffix). Mipomersen, the second antisense oligonucleotide drug approved by the FDA in 2013, is used for the treatment of homozygous familial hypercholesterolemia. Mipomersen binds to the mRNA sequence of apolipoprotein B-100 (ApoB-100) and cleaves its sequence^46^ (Fig. 3b). Because ApoB-100 is the main component of low-density lipoprotein and its precursor very low-density lipoprotein (VLDL), mipomersen can be used as a lipid-lowering medication. The backbone of these antisense oligonucleotides uses a phosphorothioate linkage, which makes them more resistant to nuclease-mediated degradation^39^.

The second group of antisense oligonucleotide drugs is primarily used to regulate the splicing of pre-mRNAs via a steric hindrance-based mechanism (Fig. 3a). Diverse RNA binding proteins affect splicing via their binding of specific sequences within the pre-mRNA transcripts, where they modulate other splicing factors to produce numerous modes of alternative splicing^47,48^. This second group of antisense oligonucleotides drugs targets these sequences in pre-mRNAs, where alternative splicing may result in the inhibition of disease. The ability to modulate this alternative splicing is unique to antisense oligonucleotides, making them a potentially valuable source of therapeutics for a variety of inherited diseases. Examples of FDA-approved antisense oligonucleotide drugs designed to modulate the splicing of their target pre-mRNAs include nusinersen, eteplirsen, and golodirsen.

Patients with spinal muscular atrophy, a rare neuromuscular disorder, have mutations in the *SMN1* gene, which encodes the survival motor neuron (SMN) protein^49^ (Fig. 3c). These mutations prevent the expression of a functional protein from the *SMN1* gene locus, and although a small amount of SMN protein is still produced from the *SMN2* gene, this protein product is smaller and less stable due to the exon skipping associated with the SMN2 pre-mRNA. Consequently, these patients experience a loss of motor neurons and muscle wasting. We also know that the intronic splicing enhancer between exons 7 and 8 in SMN2 pre-mRNA induces exon 7 skipping^49^. Nusinersen is an antisense oligonucleotide drug designed to bind to this element and block its recognition by the relevant splicing factors^50^. This blockage means that SMN2 pre-mRNA is spliced like SMN1 pre-mRNA, producing more stable SMN protein and exerting a therapeutic effect (Fig. 3c). The backbone of nusinersen also uses a phosphorothioate linkage, increasing the half-life of this drug^39^.

Both eteplirsen and golodirsen are antisense oligonucleotide drugs used in the treatment of Duchenne muscular dystrophy (DMD), a severe type of muscular dystrophy. In patients with DMD, the mRNA encoding the dystrophin protein usually contains a mutation that causes an alteration within the reading frame^51^^,^ producing a nonfunctional protein in these patients. Some patients with DMD present with a specific mutation in exon 51 of the dystrophin gene, which causes a frameshift that terminates the production of this protein (Fig. 3d). Eteplirsen binds to the exonic splicing enhancer in exon 51, which is an essential element for the inclusion of this exon in the mature mRNA^52^, resulting in exon skipping and the production of a truncated but functional version of dystrophin in these patients (Fig. 3d). Golodirsen uses a similar mechanism to induce exon 53 skipping to produce a functional version of this protein in a different set of DMD patients^53^. Both eteplirsen and golodirsen have a neutral charge and are composed of phosphorodiamidate morpholino oligonucleotides, which makes them more resistant to nuclease-mediated degradation^39^, improving their therapeutic half-life.

In addition to these approved drugs, various clinical trials using antisense oligonucleotides are currently underway for several neurological diseases that have proven to be difficult to treat using more conventional therapies. These include IONIS-HTTRx, which targets the mutant form of the huntingtin protein in Huntington’s disease^54^, and tofersen, which targets the SOD1 protein, a frequently mutated protein in a familial form of amyotrophic lateral sclerosis^55^. The preliminary data for these drugs suggest that antisense oligonucleotide-based therapeutics may provide a rich source of novel therapies for previously difficult-to-treat diseases.

### Small interfering RNAs

**Fig. 4:**
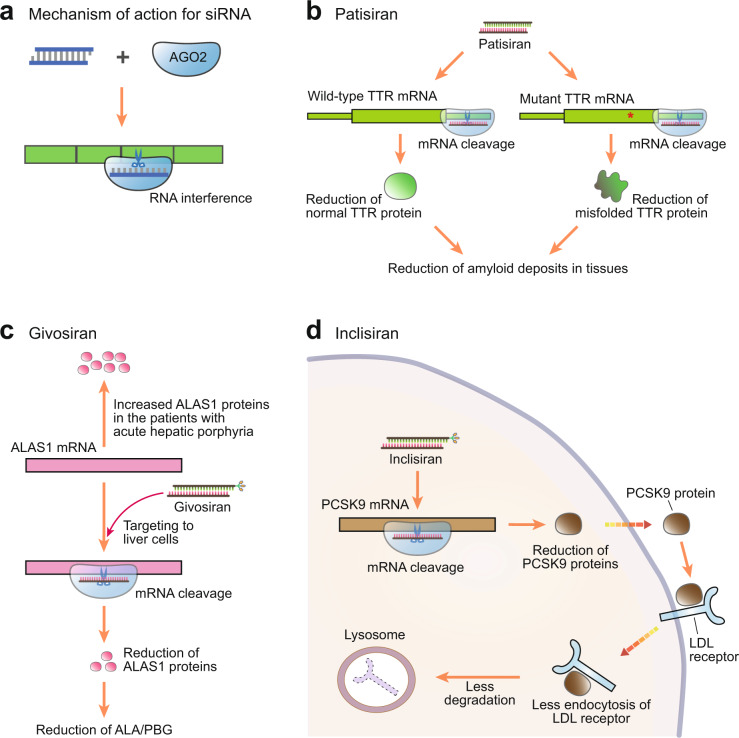
Small interfering RNA (siRNA) drugs. **a** Mechanism of action of siRNA drugs in mammalian cells. siRNAs interact with the Argonaute (AGO) protein to produce a binding complex that recognizes its target mRNA and then induces its sequence-specific cleavage in a process called RNA interference (RNAi). **b** Patisiran binds to the 3ʹ untranslated region (UTR) of transthyretin (TTR) mRNA. Because mutation occurs within the coding region of this gene, patisiran suppresses the expression of both wild-type and mutant TTR mRNAs. **c** Givosiran suppresses the expression of aminolevulinic acid (ALA) synthase 1 (ALAS1) mRNA. This results in a reduction in ALAS1 protein, which is required for the production of ALA and porphobilinogen (PBG). Notably, givosiran contains the N-acetylgalactosamine (GalNAc) conjugate within the 3ʹ end of its passenger strand. **d** Inclisiran induces the cleavage of the mRNA encoding proprotein convertase subtilisin/kexin type 9 (PCSK9), a promising target for the therapeutic management of patients with high cholesterol. Reductions in PCSK9 protein result in reduced endocytosis of low-density lipoprotein (LDL) receptors.

siRNAs use the endogenous RNAi pathway to modulate the expression of their target RNAs. In native RNAi, endogenous small RNAs form a complex with Argonaute (Ago) protein to produce an RNA-induced silencing complex (RISC), which then suppresses the expression of their target mRNAs via sequence-specific binding^56^. siRNA-based drugs use this same machinery to exert a similar effect. siRNA duplexes join the native RNAi pathway following their incorporation into RISC, where only the guide strand is retained by the Ago protein while the passenger strand is discarded (Fig. 4a). The guide strand then focuses RISC activity against its target.

To date, three siRNA-based drugs have been approved by the US FDA, patisiran, givosiran, and lumasiran (siRNA drugs are generally identified via the addition of -siran to their name). Patisiran was the first FDA-approved siRNA-based drug in 2018 and was developed to treat hereditary transthyretin-mediated (hATTR) amyloidosis^18^. This disease is caused by mutations in the transthyretin (TTR) gene, which results in the production of misfolded TTR proteins and thus in the accumulation of amyloid deposits^57^. Patisiran uses an LNP-based delivery system and is injected into the body through intravenous infusion. These particles are expected to associate with apolipoprotein E (ApoE) and enter hepatocytes through ApoE receptors^58^. Once released into the cytoplasm, patisiran is loaded into RISC and binds to the 3′ untranslated region (UTR) of both wild-type and mutant TTR mRNA, leading to the suppression of TTR protein translation and an overall reduction in amyloid deposits (Fig. 4b).

Givosiran was the second siRNA approved by the US FDA and is used in the treatment of acute hepatic porphyria^59^. This rare disease is caused by an increase in the plasma levels of aminolevulinic acid (ALA) and porphobilinogen (PBG), which are both intermediates in the heme biosynthesis pathway^60^. Givosiran targets ALA synthase 1 (ALAS1), which is the key enzyme required for the production of ALA and PBG, thus inhibiting their expression and returning heme biosynthesis to normal levels (Fig. 4c). Givosiran is delivered via a trivalent N-acetylgalactosamine (GalNAc) conjugate attached to the 3ʹ end of its passenger strand. This means that givosiran can be delivered by subcutaneous application and is targeted to the hepatocyte-specific asialoglycoprotein receptor via its GalNAc conjugate^61^. Since this method is very effective in delivering siRNA to the liver, most current liver-targeting siRNAs are being delivered using a similar method.

Inclisiran was approved by the US FDA in 2021 and is used in the treatment of primary hypercholesterolemia or mixed dyslipidemia^62^. Thus, unlike other antisense oligonucleotide or siRNA drugs, which were designed to treat the rare diseases described above, inclisiran was specifically developed to treat a broad patient group. This siRNA also uses GalNAc conjugates to mediate its delivery and suppresses the translation of PCSK9 mRNA in the liver. Because PCSK9 induces the endocytosis and degradation of the low-density lipoprotein (LDL) receptor^63^, inclisiran treatment directly reduces LDL cholesterol in the bloodstream (Fig. 4d). One of the most important characteristics of inclisiran is that its effects are very long lasting, with data showing that even a single dose of inclisiran mediates a persistent decrease in PCSK9 expression and thus reduces LDL levels for more than six months^41^. This type of reduction in the administration cycle may improve compliance, reduce costs, and decrease toxicity, highlighting another key strength of these types of therapies.

Accumulating evidence suggests that siRNA-based therapeutics may have other utilities, which explains why this remains a highly active field of research. Given these examples, we can expect to see increasing numbers of siRNA-based therapies in clinical practice in the future.

### Aptamers

**Fig. 5:**
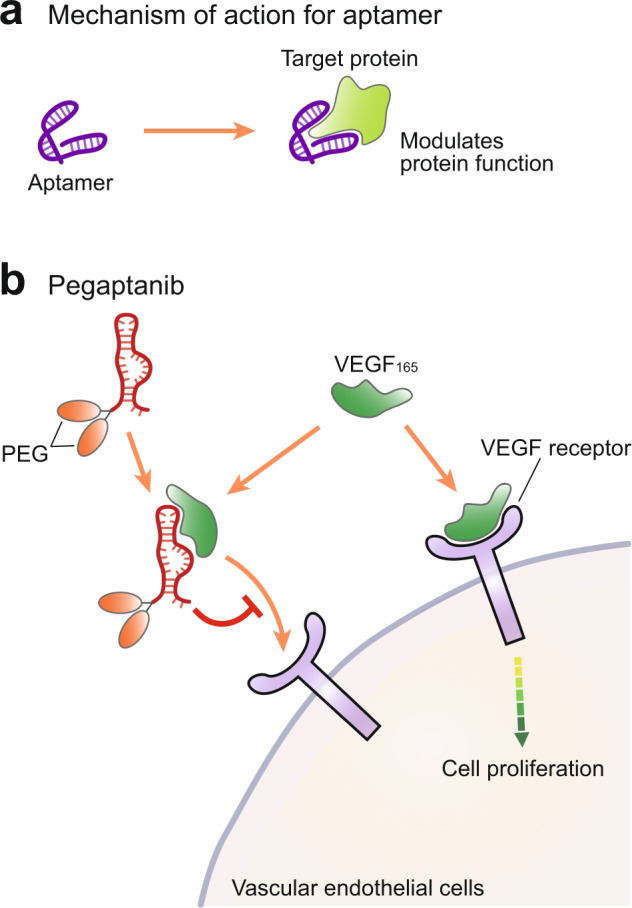
RNA aptamer drugs. **a** The mechanism of action of aptamer-based drugs. Aptamers bind to their target protein, thus modulating their function. **b** The mechanism of action of the RNA aptamer pegaptanib, which binds to vascular endothelial growth factor (VEGF), thereby inhibiting its interaction with the VEGF receptor and inhibiting cell proliferation.

Aptamers are nucleic acid constructs designed to bind specific proteins to modulate their function (Fig. 5a). To date, only one RNA-based aptamer drug (aptamers are named by adding the -apt- term) has been approved by the US FDA. Pegaptanib is a 28-nucleotide construct with two polyethylene glycol (PEG) moieties attached to its end^64^. This aptamer is designed to bind to the 165 isoform of vascular endothelial growth factor (VEGF), blocking its interaction with its receptor protein and thereby suppressing cell proliferation, the main downstream effect of VEGF signaling^65^ (Fig. 5b). Given this effect, pegaptanib was developed as a therapeutic agent for wet-type (neovascular) age-related macular degeneration (AMD)^64^. Although rarely used at present due to competition from various antibody-based drugs with similar efficacy, pegaptanib is a good example of how RNA-based aptamers can be used as therapeutics. Several RNA-based aptamers are under development, and additional aptamer-based therapeutics are expected to be available in the future^66,67^.

### Messenger RNAs

mRNA-based therapies exert their therapeutic effect by exploiting the fact that even exogenous mRNAs can be translated into functional proteins. These mRNAs are synthesized using in vitro transcription, and a cap analog is attached to their 5′ end during the reaction to facilitate their recognition by the translational machinery in the cell^68^. However, since longer RNA sequences tend to induce Toll-like receptor (TLR)-mediated immune responses^69^, mRNAs can be difficult to deliver. These difficulties slowed their clinical application, especially in their early stages of development, but were resolved by Kariko and Weissman, who reported that this reaction could be reduced by modifying the mRNA with pseudouridine^70^.

**Fig. 6:**
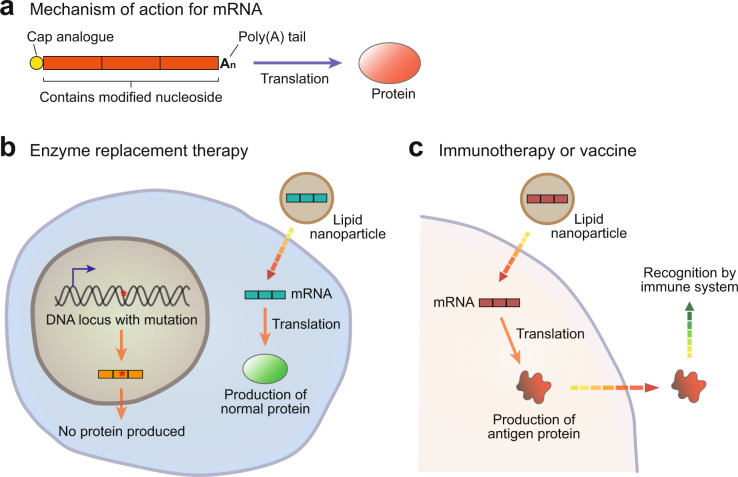
Therapeutic applications of messenger RNA (mRNA). **a** The mechanism of action of mRNA-based drugs. Exogenous mRNAs introduced into cells undergo translation to proteins and facilitate protein function. These mRNA constructs include a 5ʹ cap analog to facilitate their recognition by translation initiation factors, which is the first step in translation. In addition, mRNA sequences can be modified to allow evasion of the immune system, allowing them to exert their therapeutic effect for a longer period. **b** The use of mRNAs for enzyme replacement therapy. mRNAs are introduced into cells where the corresponding proteins are not produced due to mutation. **c** The use of mRNAs as vaccines. The introduced mRNAs produce proteins that may be recognized by the immune system as antigens.

mRNA-based therapies can be divided into two broad subcategories based on their purpose (Fig. 6a). In the first category, exogenous mRNAs are introduced into cells to replace or supplement endogenous proteins. One example of this is the treatment of patients with a genetic deficiency in an essential enzyme, and mRNA therapy can be applied to replenish the levels of this enzyme and rescue the deficiency (Fig. 6b). For example, one group used methylmalonyl-CoA mutase (MUT)-expressing mRNA to treat methylmalonic acidemia caused by a deficiency in the MUT enzyme in mice^71^. In the second category, the mRNA transcript is designed to act as a vaccine against infectious diseases or cancer antigens (Fig. 6c). Examples include the mRNA rabies vaccine described earlier^29^ and several other mRNA vaccines targeting various RNA viruses, including influenza, which are currently under development^72^.

However, the utility of mRNA-based vaccines has been convincingly demonstrated in their application as vaccines against COVID-19. Because COVID-19 caused a global pandemic over a very short period, the scientific community needed to develop an effective vaccine as soon as possible. Among the various types of vaccines, mRNA vaccines have the advantage of a relatively short development cycle. In addition, because mRNA vaccines have been under development for so long, the existing knowledge base underlying their development was robust and easily adapted to this question. This meant that scientists were able to develop two highly effective mRNA vaccines against COVID-19 in a relatively short period, less than a year after the release of the genome sequence for this virus in early 2020^37,38^. In addition, the nearly three decades’ worth of efficacy and safety data underlying the use of mRNAs in a therapeutic context supported their rapid approval by health administrations around the world. These two mRNA vaccines, tozinameran (BNT162b2, Pfizer–BioNTech vaccine) and elasomeran (mRNA-1273, Moderna vaccine), produce a modified spike protein from COVID-19, which, when administered via intramuscular injection, enters cells and facilitates spike-protein antigen presentation, allowing the maturation of a SARS-CoV-2-specific immune response in recipients.

mRNA vaccines can also be used as personalized medicines for targeting specific tumors. Here, clinicians will first identify a unique tumor mutation that is then included in an mRNA transcript. This exogenous transcript will then express this mutation, inducing a specific immune response targeting cells expressing these antigens, allowing the targeted destruction of these cells within the body. There was a recent report detailing this kind of application in the treatment of melanoma^73^, which suggested that these mRNAs can train the immune system to identify melanoma-specific mutations and eliminate the cells that express these mutant proteins, potentially reducing tumor size and preventing metastasis.

Given the likely increase in circulation of novel infectious diseases in the future and the obvious need for rapid, cost-effective solutions to prevent their spread, there is a strong argument that mRNA vaccines will become a critical weapon in these outbreaks. In addition, mRNA-based drugs are likely to be incredibly effective in treating genetic diseases that cause some form of protein deficiency. Given this, it is likely that we will continue to see the maturation of these therapeutics in the future.

## Delivery

**Fig. 7:**
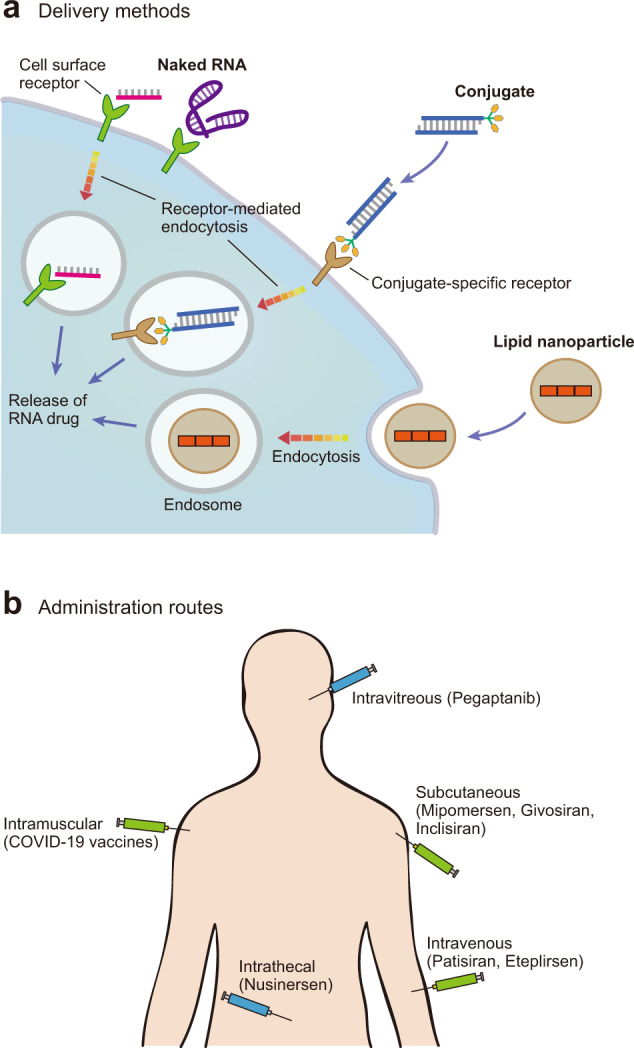
Delivery methods and administration routes for RNA-based drugs. **a** The three major delivery methods for RNA-based therapeutics in mammalian cells. Naked RNAs can be recognized by receptors that are broadly expressed across various cell types, or they can be conjugated with a compound recognized by a specific receptor. In both cases, the RNAs are introduced into the cells through receptor-mediated endocytosis. Longer RNAs are usually encapsulated inside lipid nanoparticles and endocytosed into the cells. Finally, they are released into the cytoplasm, where they exert their therapeutic effect. **b** Diverse administration routes for approved RNA-based therapeutics. RNA drugs introduced via the intravitreal or intrathecal injection require less consideration for specific delivery, as these organs are relatively severed from circulation.

The delivery of RNA-based drugs remains one of the single largest challenges for these kinds of therapies. Most delivery methods for these RNA-based therapeutics can be categorized as the addition of targeting moieties, the encapsulation of RNAs into lipid-based nanoparticles, and direct delivery into the target organ without extensive modification (Fig. 7a).

In the case of most GalNAc-conjugated siRNAs, such as givosiran and inclisiran, the GalNAc moiety is attached to the 3ʹ end of the passenger strand of the siRNA^59^, allowing recognition by the asialoglycoprotein receptor, a protein that is primarily associated with hepatocytes^61^, resulting in targeted delivery to the liver. The increased efficiency of this method has largely displaced the LNP-based delivery of small RNA drugs to the liver. In fact, the Alnylam company, which developed the first siRNA drug, patisiran, for the treatment of amyloidosis, designed this drug to use the LNP-based delivery method but is now testing their new siRNA drug, vutrisiran, which was engineered to use GalNAc-based delivery, for treating the same disease^74^. However, no other specific conjugates for cell-specific delivery have been evaluated or approved following the clinical trial. The success of GalNAc-based delivery supports the need for more research on conjugate-mediated cell-specific delivery.

RNAs encapsulated in LNPs are significantly protected against nuclease-mediated degradation. However, the LNP itself can be toxic and may induce immunostimulatory side effects^75^. Nevertheless, the delivery of RNA-based drugs comprised of longer sequences, such as mRNA-based therapies, still relies on LNPs or other similar materials. Although the need to target a specific organ is relatively low when using a mRNA vaccine, delivery to the desired organ is important when using mRNA for the purpose of treating enzyme deficiencies, which can be made more difficult by the fact that LNPs are primarily transported to and eliminated in the liver or kidney^76^. This is why many of the current RNA-based therapies focus on diseases in these organs. Therefore, future work in this field should focus on expanding the delivery of these constructs to other organs and cell types.

Finally, many studies have evaluated the use of naked RNA delivery when the target is suitable for direct RNA delivery. Some examples of this include nusinersen, which is administered via intrathecal injection, and pegaptanib, which is administered via intravitreal injection (Fig. 7b). In both cases, this method of administration is possible because it confines these drugs to their targeted organ, making them unlikely to be filtered out or eliminated by the liver or kidney. The success of these drugs suggests that this delivery method may be feasible for treating some eye and neurological diseases.

In summary, different delivery methods are used based on the available material and target. However, the drugs in the class of RNA-based therapies are significantly larger than those in other therapeutic classes, including small molecules, which makes their delivery more complex. This is exacerbated by the native charge on these compounds, which may inhibit their movement across the cell membrane. Therefore, more research on delivery methods for RNA-based drugs is needed.

## Conclusion

This review presents a solid case for the use of RNA-based drugs in the treatment of various diseases, especially those that are difficult to treat using other drug types. This is particularly clear considering the benefits of personalized RNA drugs, which may open up the possibility of high-efficiency treatment for very rare diseases for which conventional treatments have not been developed due to their cost. Moreover, RNA technology has mediated the rapid development of particularly efficient vaccines over the last couple of years, thus highlighting their utility in combating future pandemics and other infectious diseases. Although their delivery to target organs and their efficient introduction into cells remain challenging, the many advantages of RNA drugs (including their potential to target a wide range of genetic molecules, fast and efficient production, long-term effect, usefulness for rare diseases and reduced risk of genotoxicity) make the development of these technologies a worthwhile investment. Inspired by the development of various novel RNA drugs, including the novel COVID-19 vaccines in 2020, many researchers are making unprecedented efforts to develop new RNA-based drugs. Therefore, with the advancement of RNA therapy technology, the development of more diverse RNA-based drugs and drug-delivery methods is expected in the future.
